# Estimation of best corrected visual acuity based on deep neural network

**DOI:** 10.1038/s41598-022-22586-2

**Published:** 2022-10-24

**Authors:** Woongsup Lee, Jin Hyun Kim, Seongjin Lee, Kyonghoon Kim, Tae Seen Kang, Yong Seop Han

**Affiliations:** 1 Department of Information and Communication Engineering, Gyeongsang National University, Tongyeong, Republic of Korea; 2 Department of AI Convergence Engineering, Gyeongsang National University, Jinju, Republic of Korea; 3 School of Computer Science and Engineering, Kyungpook National University, Daegu, Republic of Korea; 4 Department of Ophthalmology, Gyeongsang National University Changwon Hospital, #11 Samjeongja-ro, Seongsan-gu, Changwon, 51472 Republic of Korea; 5 Department of Ophthalmology, Gyeongsang National University College of Medicine, Institute of Health Sciences, Jinju, Republic of Korea

**Keywords:** Computational biology and bioinformatics, Anatomy, Health care, Medical research

## Abstract

In this study, we investigated a convolutional neural network (CNN)-based framework for the estimation of the best-corrected visual acuity (BCVA) from fundus images. First, we collected 53,318 fundus photographs from the Gyeongsang National University Changwon Hospital, where each fundus photograph is categorized into 11 levels by retrospective medical chart review. Then, we designed 4 BCVA estimation schemes using transfer learning with pre-trained ResNet-18 and EfficientNet-B0 models where both regression and classification-based prediction are taken into account. According to the results of the study, the predicted BCVA by CNN-based schemes is close to the actual value such that 94.37% of prediction accuracy can be achieved when 3 levels of difference can be tolerated during prediction. The mean squared error and $R^2$ score were measured as 0.028 and 0.654, respectively. These results indicate that the BCVA can be predicted accurately for extreme cases, i.e., the level of BCVA is close to either 0.0 or 1.0. Moreover, using the Guided Grad-CAM, we confirmed that the macula and the blood vessel surrounding the macula are mainly utilized in the prediction of BCVA, which validates the rationality of the CNN-based BCVA estimation schemes since the same area is also exploited during the retrospective medical chart review. Finally, we applied the t-distributed stochastic neighbor embedding to examine the characteristics of CNN-based BCVA estimation schemes. The developed BCVA estimation schemes can be employed to obtain the objective measurement of BVCA as well as the medical screening of people with poor access to medical care through smartphone-based fundus imaging.

## Introduction

Vision plays a significant role in our daily lives and good eye condition can promote economic opportunities, improve quality of life^1^, and enable better educational outcomes^2^. Approximately 596 million people had distance vision impairment worldwide, of whom 43 million were blind by 2020^3^, and the majority of whom live in middle- or low-income countries with poor access to eye care. Given that over 80% of such vision impairments can be avoidable through early detection and treatment, it is important to have regular eye health screening.

Best-corrected visual acuity (BCVA) is the measurement of the possible ability to distinguish shapes and the details of objects at a given distance with corrective lenses, and is one of the most commonly used testing factors for eye conditions. The accurate measurement of BCVA is important because clinicians depend on these results to determine further investigations and quantify changes to vision over time^1^. The most common way to measure BCVA is to use a chart, such that the patient is asked to identify letters on the chart^4–6^, e.g., Snellen or Landolt C chart. However, such manual BCVA estimations rely on the response from the patients, making them ineffective when the patients are unable to respond. Moreover, manual approaches can be time-consuming and can be subjective to factors such as the BVCA measuring environment. To deal with these shortcomings, objective automatic BCVA estimation schemes were developed, where the BCVA level is predicted-based on various factors such as the visual evoked potential (VEP)^5,7^ or the optical coherence tomography angiograsphy (OCTA)^8^.

Recently, deep learning, based on the deep neural network (DNN), has gained great popularity due to its significant performance gains over conventional schemes^9–11^. Especially, the classifications based on the convolutional neural network (CNN), which is specialized in extracting spatial features, are shown to achieve far more accurate performance than the conventional handcrafted schemes based on the analytic models and even surpassed human-level performance^12,13^. As a result, CNN-based schemes have been investigated extensively in the diagnosis based on medical images, including head computed tomography (CT), lung CT and fundus image^14^. Specifically, for the application of CNN in the analysis of head CT, brain stroke detection and lesion segmentation were mainly examined^15–17^, whereas the detection of lung cancer^18–20^ or the diagnosis of COVID-19^21–24^, were mainly taken into account in the analysis of lung CT.

In this study, we estimated BCVA levels using different types of CNNs; where the BCVA level is predicted by observing a fundus image that involves photographing the rear of an eye. The motivation behind using the fundus image is that it is most popularly used in examining eye diseases, and the advent of smartphone-based fundus imaging^25–28^ makes it easy to obtain fundus images. Moreover, in recent days, deep learning has been extensively applied to the fundus image to detect the lesion and diagnose eye-related diseases such as macular degeneration and diabetic retinopathy (DR)^29–31^. Deep learning has also been employed for the quality grading of fundus image^32^, and for the segmentation of blood vessel from fundus image^33^, which validate the applicability of deep learning for the fundus image. However, the deep learning-based estimation of BCVA levels from fundus images has been rarely investigated despite its importance in practice, such that the application of deep learning for estimating BCVA levels from fundus images is the contribution of our work. Moreover, we verified the performance of the proposed scheme using a large number of actual data, i.e., 53,318 fundus images, collected at the Gyeongsang National University Changwon Hospital, and showed that the BCVA level could be predicted with high accuracy, which is another contribution of our work. We also used class activation visualization, i.e., Guided Grad-CAM^34^, to confirm the operation of CNN-based BCVA estimation schemes, and the characteristics of fundus images for each BCVA level were verified using t-distributed stochastic neighbor embedding (t-SNE) clustering^35,36^, both of which are another contribution of our work. We note that unlike manual BCVA measurement, which is affected by the BCVA measuring environment, the proposed methodology can provide an objective measurement of BCVA, which can be used to determine the necessity of other expensive and time-consuming BCVA measurements such as VEP, electroretinography (ERG) or multifocal-ERG (mfERG). Moreover, our BCVA measurement can be helpful in the analysis of clinical studies of ophthalmology. Finally, it can be employed in public health facilities to provide efficient ways of eye health screening for people who have poor access to public health. Especially, the proposed methodology can be employed in a mobile application so people can self-measure their BCVA level using the smartphone conveniently^25–28^.

## Results

### Characteristics of collected fundus dataset

The fundus images of patients who visited Gyeongsang National University Changwon Hospital, South Korea, from February 2016 to December 2020, were collected, and the fundus images from the left and right eyes were utilized altogether. Each collected fundus image was examined through retrospective medical chart review and labeled into 11 levels ranging from 0.0 to 1.0. We note that the case of no light perception (LP-), light perception (LP), hand movement (HM), and decimal VA scale 0.01 and 0.025 that correspond to Snellen VA scale of 20/2000 and 20/800, is counted as BCVA level of 0.0. It is also worth noting that we used the same BCVA level that the expert ophthalmologists use at the Gyeongsang National University Changwon Hospital so that the entire BCVA levels can be covered. In total, 53,318 fundus images were accumulated where the number of fundus images which corresponds to BCVA level of 0.0, 0.1, 0.2, 0.3, 0.4, 0.5, 0.6, 0.7, 0.8, 0.9, and 1.0 is 1760, 1153, 1711, 2175, 2061, 3386, 3969, 4524, 4553, 6454, and 21572, respectively. Moreover, 90% of these collected fundus images were used for training CNN models, while the rest 10% of fundus images were used for validation.

### Considered schemes and performance metrics

As an underlying pre-trained CNN structure, we considered two CNN structures, ResNet-18^37^ and EfficientNet-B0^38^, and both were pre-trained with ImageNet dataset^39^. Moreover, we considered both regression-based and classification-based BCVA estimation schemes, where, the level of BCVA is estimated as a continuous value in the former case while it is predicted as discrete levels in the latter case. The classification-based BCVA estimation schemes using ResNet-18 and EfficientNet-B0 are denoted as *Res-Cla* and *Eff-Cla*, respectively, while regression-based BCVA estimation schemes using ResNet-18 and EfficientNet-B0 are denoted as *Res-Reg* and *Eff-Reg*.

Regarding the performance metrics, we considered the precision, which is denoted as $\frac{TP}{TP+FP}$, where *TP* and *FP* denote the true positive and false positive, respectively. In the calculation of precision, the estimated BCVA of regression-based schemes, i.e., Res-Reg and Eff-Reg, was rounded such that it can have a discrete value. Moreover, the root mean square error (RMSE) and the $R^2$ score were taken into account as performance metrics^40^. Formally, the RMSE is expressed as ${\mathbb {E}} \left[ \left( VA - {\hat{VA}} \right) ^2 \right]$, where *VA* and ${\hat{VA}}$ are the actual and predicted BCVA levels, and $R^2$ score can be formulated as $1-\frac{\sum \left( {\hat{VA}} - VA \right) ^2 }{\sum \left( {\mathbb {E}}[VA] - VA \right) ^2 }$. We note that the RMSE will be close to 0 when the prediction is accurate whereas the $R^2$ score is close to 1 when the prediction is accurate.

**Table 1:** The precision of considered BCVA estimation schemes.

Prediction model	Res-Cla (%)	Eff-Cla (%)	Res-Reg (%)	Eff-Reg (%)
No relaxation	**44.28**	37.05	35.91	30.18
1 level relaxation	69.84	71.01	**71.11**	69.55
2 level relaxation	82.29	85.01	86.69	**87.03**
3 level relaxation	89.94	92.32	94.12	**94.37**

For each relaxation case, the scheme with the highest precision is highlighted in bold font.

**Table 2:** MSE and $R^2$-score of considered BCVA estimation schemes where the highest performance is highlighted in bold font.

Prediction model	Res-Cla	Eff-Cla	Res-Reg	Eff-Reg
RMSE	0.042	0.036	**0.028**	0.029
$R^2$-score	0.501	0.575	**0.654**	0.648

### Performance comparison of considered schemes

Table 1 shows the precision of considered BCVA estimation schemes. For the precision calculation, we assumed that a certain level of discrepancy could be tolerable, which is expressed by the level of relaxation. For example, when 1 level relaxation is used and the actual BCVA level is 0.5, our prediction is assumed to be correct when the predicted BCVA level is either 0.4, 0.5, or 0.6. Without relaxation, Res-Cla showed the highest precision whose value is 44.28%, while Res-Reg achieved the highest precision value for 1 level relaxation, i.e., 71.11%. Moreover, for 2- and 3-level relaxations, Eff-Reg showed the highest precisions of 87.03% and 94.37%, respectively. Table 2 shows the RMSE and $R^2$-score, where the Res-Reg achieved the lowest RMSE of 0.028 and the highest $R^2$-score of 0.654. Moreover, the Eff-Reg achieved almost the same performance as the Res-Reg, which is much higher than that of classification schemes.

### Classification results using confusion matrix

**Figure 1:**
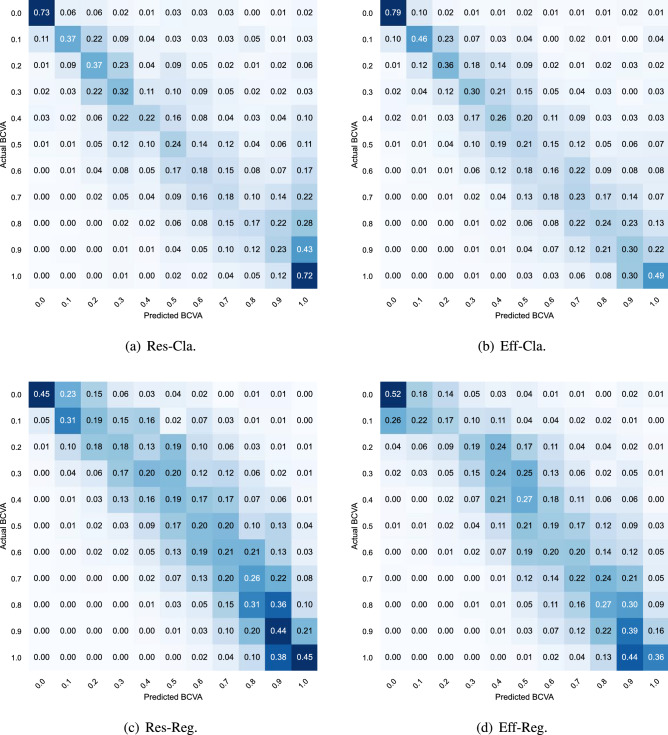
Confusion matrix of considered schemes.

Figure 1 shows the confusion matrix of the considered schemes. The elements concentrate near diagonal for all confusion matrices such that the wrong prediction result will not diverge significantly from the ground truth value. Especially, the level of concentration is higher for regression schemes (Res-Reg and Eff-Reg) than for classification schemes (Res-Cla and Eff-Cla). Moreover, the sum of diagonal elements is highest for the Res-Cla; and the classification accuracy is higher when the actual level of BCVA is near 0.0 or 1.0 than the case when it is near 0.5. The highest accuracy obtained, 79%, can be obtained for the Eff-Cla when the level of BCVA is 0.0.

### Classification results using histogram

**Figure 2:**
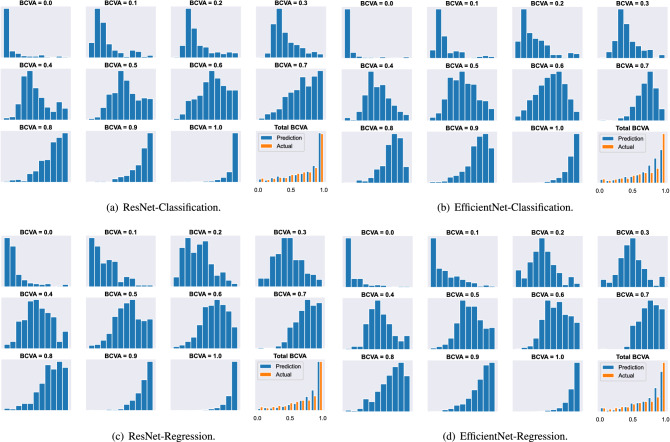
Histograms of considered schemes for each BCVA level. The last subgraph corresponds to the histogram of all prediction results and actual BCVA levels.

Figure 2 shows the histogram of the considered schemes. The prediction distribution is concentrated near the ground-truth value, such that the wrong prediction will not diverge significantly from the actual BCVA level. Moreover, the distribution concentrated more densely when BCVA = 0.0 and 1.0, which coincides with our findings observed in previous results on the confusion matrix.

### Validation of considered schemes using class activation visualization

**Figure 3:**
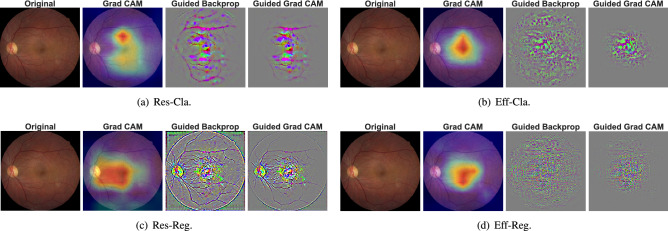
Class activation visualization of the considered schemes with fundus image. For each sub-figure, the first, second, third, and fourth image correspond to the original fundus image, Grad CAM results, Guided Back-propagation result, and Guided Grad CAM results, which combine Grad CAM and Guided Back-propagation, respectively.

Figure 3 shows the class activation visualization of the considered schemes with the corresponding fundus image, where the Grad-CAM, Guided Back-propagation, and Guided Grad-CAM are taken into account for the visualization, where more results can be found in our data repository^41^. The actual BCVA level is 1.0, and the predicted BCVA of Res-Cla, Eff-Cla, Res-Reg, and Eff-Reg are 0.7, 1.0, 0.934, and 0.968, respectively. According to the Grad-CAM results, the area near the macula is highlighted for all considered schemes, while the optic disk is also highlighted for the Res-Reg. For the Guided Back-propagation, the blood vessel, macula, and optic disk are highlighted. Finally, for the Guided Grad-CAM, the blood vessel and macula are highlighted, which coincides with the area inspected during the retrospective medical chart review to identify BCVA.

### Samples of wrong prediction result

**Figure 4:**
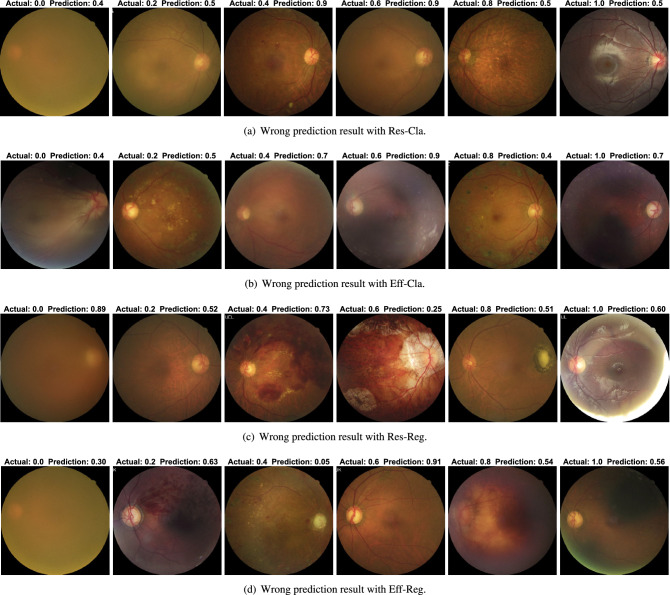
Wrong prediction result of the considered scheme when actual BCVA areis 0.0, 0.2, 0.4, 0.6, 0.8, and 1.0. The actual and predicted BCVA is indicated at the top of the figure.

Figure 4 shows the randomly selected samples of wrong prediction results for all the considered schemes when the actual BCVA is 0.0, 0.2, 0.4, 0.6, 0.8, and 1.0. Some of the wrong prediction results are caused by the inappropriate fundus image. For example, macula and optic disc could not be identified properly from the fundus images for the case when the actual BCVA is 0.0, which makes the identification of BCVA challenging.

### Validation of considered schemes using class activation t-SNE

**Figure 5:**
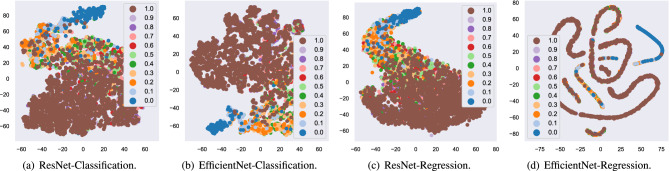
T-SNE result of considered schemes.

Figure 5 shows the clustering results using t-SNE. Although data points for each BCVA level overlap to some extent, they are aligned monotonically according to their corresponding BCVA level in general. Additionally, data points for the case when BCVA is 0.0 can be clustered separately, which suggests that this case can be classified accurately.

## Discussion

In general, the level of BCVA is measured through examination using the Snellen or Landolt C chart. However, when a patient is unconscious due to brain damage or a disease such as dementia, it will be impossible to determine the level of BCVA. Moreover, such manual BVCA measurement cannot apply to infants and childhood. Furthermore, some patients give false response to the examination to get a high disability rating. In such a case, the measurement of BCVA level through the direct inspection of the fundus is necessary, where the macula, major retinal arteries, and veins are used for the examination.

Recently, CNN has been applied extensively in retinal fundus image analysis. Specifically, CNN-based detection and classification of DR, which is a diabetes complication causing blindness, has been investigated^42–45^. Moreover, a study confirmed that the CNN-based approach could be used for the estimation of gender from retinal fundus image^46^ and the identification of left and right fundus image^47^. Since the spatial feature of fundus images can be extracted by CNNs, we examined the feasibility of CNN-based BCVA estimation in this study.

Table 1 shows the precision of considered CNN-based BCVA estimation schemes, i.e., Res-Cla, Eff-Cla, Res-Reg, and Eff-Reg. Unlike conventional classification tasks, the severity of misclassification can differ depending on the misclassified results. For example, when the actual BCVA level is 0.1, the prediction of 0.2 can be regarded as more accurate than prediction of 0.9, even though both prediction results are incorrect. It is worth noting that the prediction results obtained through the retrospective medical chart review can be slightly different for the same fundus image, and such minor deviation is usually acceptable in practice. To properly reflect such characteristics in the misclassification, we evaluated the precision by allowing such a small difference in the prediction, namely the level of relaxation. Accordingly, when 2 level relaxation is adopted, we assumed that prediction is correct if $|{\hat{VA}} - VA| \le 0.2$ where *VA* and ${\hat{VA}}$ are actual and predicted BCVA levels.

As shown in Table 1, the classification schemes achieve higher precision than the regression schemes when the relaxation is not adopted. On the other hand, the regression schemes provide higher accuracy when the relaxed precision is adopted. We conjecture that the utilization of the different loss functions causes such a phenomenon. More specifically, we train the classification schemes to exactly match the predicted BCVA to the actual BCVA, whereas the regression schemes are trained to minimize the difference between predicted and actual BCVA levels. Accordingly, classification schemes are better at finding the exact BCVA level while the prediction of regression schemes can be closer to its actual value when the predicted BCVA level is different from the actual value. Moreover, the Res-Cla achieves the highest precision of 44.28% for no relaxation case, whose value is somewhat low for practical uses. However, as 1, 2, and 3 levels of relaxations are adopted, the achievable precision can increase to 71.11% (Res-Reg), 87.03% (Eff-Reg), and 94.37% (Eff-Reg), respectively. It is worth noting that the manual BVCA measurement is subjective, and the measurement of BVCA can change depending on the measuring environment and time. Accordingly, a minor difference in BCVA prediction is generally acceptable such that we can conclude that our considered BCVA estimation schemes can be handy in practical usage.

Table 2 shows the RMSE and $R^2$-Score of considered BCVA estimation schemes. The regression schemes provide better performance than the classification schemes because we trained the regression schemes to minimize the difference between the actual and predicted BCVA level, as explained previously, whereas this difference determines the RMSE and $R^2$-Score. Specifically, the Res-Reg scheme achieves the highest performance (i.e., lowest RMSE and highest $R^2$-Score) while the Eff-Reg scheme achieves almost the same performance as the Res-Reg scheme.

Next, to better understand the prediction accuracy for each BCVA level, we showed the accuracy of considered BCVA estimation schemes using the confusion matrix as depicted in Fig. 1. The diagonal element corresponds to the accuracy for each BCVA level. As can be observed from confusion matrices, the diagonal elements are generally small. The diagonal values are smaller when the BCVA level is close to 0.5 compared to cases when the BCVA level is either 0.0 or 1.0. For example, the accuracy of Res-Cla is 79% and 72% when the BCVA level is 0.0 and 1.0, respectively, while the accuracy is 24% when the BCVA level is 0.5. This is because when the BCVA level is either 0.0 or 1.0, the fundus image contains the unique characteristic, which is easy to identify. However, as the BCVA level approaches 0.5, such a unique characteristic becomes ambiguous, making the identification of BCVA more challenging. Also, examinations by expert ophthalmologists are more confident when BCVA levels are either 0.0 or 1.0. Among all considered schemes, the trace of the confusion matrix is highest for Res-Cla, i.e., highest accuracy, which is in line with the performance evaluation in Table 1.

Although the diagonal elements are not dominant in the confusion matrix, the values near the diagonal are considerably large. This means that even when the predicted BCVA level is incorrect, the difference between the wrong prediction result and the actual BCVA level is not significant. Also, the level of concentration to the diagonal element is higher for regression schemes (i.e., Res-Reg and Eff-Reg) than for classification schemes (i.e., Res-Cla and Eff-Cla). Thereby, regression schemes will be more accurate, which coincides with our conclusion in Table 2. Moreover, we also found only a minor difference in performance according to baseline CNN models, i.e., ResNet-18 and EfficientNet-B0.

Figure 2 depicts the histogram of the estimated BCVA level for all considered schemes. The distribution of the predicted BCVA level concentrates around the actual BCVA level, i.e., the wrong prediction will be close to the ground-truth value, and the distribution becomes more densely concentrated when BCVA is 0.0 and 1.0, which agrees with our previous observation in Fig. 1. Moreover, the distribution of classification schemes is also concentrated around the actual BCVA level even though we do not consider the difference between the prediction and the ground-truth BCVA level during the training. To be more specific, we adopted the cross-entropy loss function for the training of DNN in classification schemes. Thereby, the value of the loss function will be the same for wrong predictions. For example, when the actual BCVA level is 0.2, the inaccurate prediction of 0.3 will not have a lower value of loss function than the case with a wrong prediction of 1.0. Consequently, the training will not force the prediction results to locate near the actual BCVA level. According to the results, the similarity exists in fundus images for nearby BCVA levels, e.g., the spatial features of the fundus image corresponding to the BCVA level of 0.2 will be similar to those corresponding to the BCVA level of 0.3, as will be confirmed later using the clustering with t-SNE.

Due to the black-box nature of DNN^9^, it is hard to justify why such a prediction on the BCVA level is obtained from the fundus image, which is one of the main drawbacks of CNN-based schemes. To better understand the operation of the considered CNN-based schemes, we applied Guided Grad-CAM, which combines Grad-CAM and Guided Back-propagation to identify the dominant spatial features for the prediction. Figure 3 depicts the original fundus image and the resulting Grad-CAM, Guided Back-propagation, and Guided Grad-CAM overlaid on the original fundus image for all considered schemes, where the actual BCVA level is 1.0, and the prediction of Res-Cla, Eff-Cla, Res-Reg, and Eff-Reg are 0.7, 1.0, 0.934, 0.968, respectively. The area near the macula is highlighted for all considered schemes in the Grad CAM. On the other hand, the blood vessel, macula, and optic disk are emphasized for the Guided Back-propagation result. As a consequence, for the Guided Grad-CAM, the macula and blood vessel surrounding the macula are highlighted, i.e., BCVA estimation schemes make their predictions by observing these features. Since the same spatial features are used in the retrospective medical chart review to identify BCVA levels, we can conclude that the prediction of the considered BCVA estimation schemes is reasonable and trustworthy.

To further analyze the inaccurate prediction results, Fig. 4 presents the randomly selected samples of wrong prediction results for all considered schemes, when the actual BCVA is 0.0, 0.2, 0.4, 0.6, 0.8, and 1.0. In some cases, wrong prediction results are obtained due to the inappropriate fundus images. For example, the macula and optic disc could not be identified from the fundus images for the case when the actual BCVA was 0.0. Moreover, for Eff-Cla with BCVA level of 1.0 and Eff-Reg with BCVA level of 1.0, the macula is shaded and hard to recognize, which makes the identification of BCVA levels more challenging.

Finally, to investigate the correlation of fundus images for each BCVA level, Fig. 5 depicts the visualization result using t-SNE. Data points are aligned monotonically according to their corresponding BCVA. This result reveals that similarity exists in spatial features for fundus images according to their BCVA levels. Additionally, the data points for the BCVA level of 0.0 can be grouped separately which suggests that the fundus image with a BCVA level of 0.0 can be classified accurately.

## Methods

### Study design

The protocol of this retrospective study was approved by the Institutional Review Board of Gyeongsang National University Changwon Hospital and the principles of the Declaration of Helsinki. The requirement for obtaining informed patient consent was waived by the institutional review board (GNUCH 2021-05-007) due to the retrospective nature of the study.

### Acquisition of fundus data

The fundus images of patients who visited Gyeongsang National University Changwon Hospital were obtained by an expert examiner using a digital retinal camera. The black area of the fundus image, which surrounds the retinal image, was removed through trimming. The fundus images were reviewed and labeled by expert ophthalmologists such that each fundus image can be associated with one BCVA level ranging from 0.0 to 1.0. During the acquisition of data, LP-, LP, HM, and decimal VA scale 0.01 and 0.025, which correspond to the Snellen VA scale of 20/2000 and 20/800, was labeled as BCVA level of 0.0.

### Pre-processing of data

**Figure 6:**
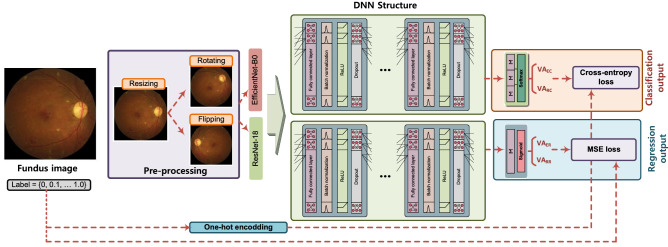
The procedure considered the BCVA estimation scheme. The fundus image was resized to $224\times 224\times 3$, and the random flipping and rotating are applied as a means of data augmentation. The modified fundus images were then fed into pre-trained ResNet-18 and EfficientNet-B0, whose outputs were fed into a DNN structure composed of a fully connected layer, batch normalization, dropout, and ReLU. The softmax function is used as a last layer for classification schemes (i.e., Res-Cla and Eff-Cla) whereas the regression schemes (i.e., Res-Reg and Eff-Reg) employ the sigmoid function instead. The parameters of the considered DNN structure are updated using cross-entropy loss and MSE loss using the level of BCVA as a label for classification schemes and regression schemes, respectively.

The fundus images were pre-processed before being fed into CNN. First, the size of the original fundus image was reduced to 224x224x3, which is necessary to reduce the computational overhead. Then, the fundus image were randomly flipped horizontally and rotated to a maximum rotation of 5 degrees. The pre-processing is necessary as a means of data augmentation because the number of fundus images is not sufficient for some BCVA levels, i.e., imbalance in data size. The considered pre-processing of the fundus image is shown in Fig. 6.

### Convolutional neural networks

In our current study, we considered two types of estimations, i.e., the classification scheme and the regression scheme, where the training methodology and output layer of the DNN structure are different for both schemes. Moreover, to expedite the training of the neural network, we employed the transfer learning (TL)^48^ such that the pre-trained baseline CNN models are connected in tandem with the DNN structure composed of fully connected layers, as shown in Fig. 6. We take into account the ResNet-18^37^ and EfficientNet-B0^38^ as baseline CNN models. The ResNet-18^37^ is a CNN structure composed of 18 layers, where the residual connection is employed to improve the performance of CNN. On the other hand, the EfficientNet-B0^38^ is a CNN structure composed of 237 layers, where the compound scaling is employed to achieve better performance with a lower number of parameters. Both ResNet-18 and EfficientNet-B0 are first pre-trained with ImageNet dataset^39^. We note that both baseline CNN models can be used for both estimations.

Then, the output of the baseline CNN model is fed into the DNN structure, which summarizes spatial features extracted from the CNN model. For the DNN structure, the batch normalization^49^ and dropout^50^ were applied, and the rectified linear unit (ReLU) was utilized as an activation function. Depending on the estimations, the last layer of the DNN structure is different. Specifically, for the classification scheme, the last layer of the DNN is connected to the softmax function, whereas the sigmoid function is adopted for the regression scheme, as shown in Fig. 6, where $VA_{EC}$, $VA_{RC}$, $VA_{ER}$, and $VA_{RR}$ denote the estimated BCVA level for Eff-Cla, Res-Cla, Eff-Reg, and Res-Reg, respectively. We note that the classification and regression schemes use different DNN structures.

Regarding the training of DNNs, only the parameters of the DNN at the latter part are updated during the training because the baseline CNN models are pre-trained with the ImageNet dataset. For the regression schemes, the mean squared error (MSE) loss was employed. On the other hand, the cross-entropy loss was used for classification schemes where the label data on BCVA levels was transformed to a vector whose length is 11, using a one-hot encoding. The trained DNN models can be found in our data repository^41^.

### Class activation visualization

The Guided Grad-CAM^34^ that combines Grad-CAM and Guided Back-propagation was adopted for the class activation visualization. In the Grad-CAM, the outputs of the final convolutional layer were summarized to find the most critical area that affects the BCVA estimation the most. On the other hand, in the Guided Back-propagation, only the positive gradients and the input signal were visualized. Finally, in the Guided Grad-CAM, by combining the results of Grad-CAM and Guided Back-propagation, we identified the main spatial features which affect the BCVA estimation most.

### Clustering using t-SNE

We used t-SNE^35,36^, which is one of the most popular nonlinear dimensionality reduction technology, to visualize the spatial features of the fundus for each BCVA level. In the calculation of t-SNE, the dimension of the output of the last layer of the DNN structure was compressed to two-dimensional space.

## Data Availability

Data supporting the findings of the current study are available from the corresponding author upon reasonable request.
